# An Investigation into the Thermomechanical Processing and Dynamic Recrystallization Mechanisms of High-Magnesium Aluminum Alloys

**DOI:** 10.3390/ma18122734

**Published:** 2025-06-11

**Authors:** Zili Ye, Zixiao Zhou, Zhaolin Ye, Zhi Wang, Qizhong Zhao, Konda Gokuldoss Prashanth

**Affiliations:** 1 National Engineering Research Center of Near-Net-Shape Forming for Metallic Materials, South China University of Technology, Guangzhou 510640, China; yezili.2000@163.com (Z.Y.); zixiaozhou11@gmail.com (Z.Z.); yzlyezhaolin@163.com (Z.Y.); kgprashanth@gmail.com (K.G.P.); 2 ALG Aluminium Inc., Nanning 530031, China; 3 Department of Mechanical and Industrial Engineering, Tallinn University of Technology, Ehitajate Tee 5, 19086 Tallinn, Estonia; 4 CBCMT, School of Mechanical Engineering, Vellore Institute of Technology, Vellore 632014, India

**Keywords:** aluminum alloy, thermal processing diagram, high-temperature deformation behavior, thermal processing

## Abstract

In this study, we studied the dual role of magnesium on the high-temperature deformation mechanisms and microstructural evolution of high-Mg 5383 aluminum alloys. We developed a quantitative framework to characterize high-temperature flow behavior and constructed 3D processing maps to identify processing instabilities. The results indicate that solid solution strengthening induced by Mg atoms leads to a substantial increase in peak flow stress. The thermal activation energy rises significantly from 182 kJ/mol to 209 kJ/mol at a Mg content of 5 wt.%, which highlights the pronounced solute drag effects on dislocations. Moreover, Mg-modified grain boundary dynamics enhance power dissipation efficiency by 34% (from 35% to 47%). With an increasing Mg content, the processing instability domains expand, thereby shifting the optimal processing parameters towards higher-temperature and lower-strain-rate regions (500 °C/0.05 s^−1^). The results provide a theoretical foundation for optimizing the thermal processing characteristics and mechanical properties of high-Mg aluminum 5xxx series alloys.

## 1. Introduction

As a lightweight engineering material, research on the thermal deformation behavior of aluminum alloys is of utmost importance for the development of engineering applications in aerospace, marine vessels, and ocean equipment [1,2,3]. Constitutive models serve as core tools for describing the relationships between the flow stress of aluminum alloys and parameters such as the temperature, strain rate, and strain. Existing research primarily focuses on the following aspects: (1) thermal deformation behavior and the establishment of constitutive models for typical aluminum alloys, (2) key influencing factors and micro-mechanisms, and (3) model comparison and optimization directions.

Charpentier [4] studied the flow behavior and softening mechanism of 2024 aluminum alloys across a wide temperature range with different strain rates. By incorporating friction and deformation heat corrections, they established a constitutive model. Experiments showed that the flow curve of 2024 aluminum alloys exhibit a “peak + softening” pattern above 250 °C, while strain hardening is observed at 145 °C. Softening is dominated by dynamic recovery, and recrystallized grains appear in local areas under high-temperature and high-strain-rate conditions. Senthil [5] conducted tensile tests on 7075-T651 aluminum alloys under different stress states, strain rates, and temperatures (25–600 °C), calibrated the Johnson–Cook model, and analyzed the anisotropy of 7075 aluminum alloys. The results indicate that stress triaxiality significantly affects strength and ductility, with increasing temperature leading to a decrease in flow stress. The Johnson–Cook model requires the introduction of Hill stress potentials to describe anisotropy, but strain rate sensitivity is not significant within the studied range. Liang Chen [6] performed thermal compression tests on 6026 aluminum alloys at 673–823 K and different strain rates, comparing the original Johnson–Cook, modified Johnson–Cook, Arrhenius, and strain-compensated Arrhenius models. They found that the strain-compensated Arrhenius model exhibits higher prediction accuracy than the original Johnson–Cook model (correlation coefficient R increased from 0.988 to 0.998). Wang [7] described the constitutive behavior of 5A06 aluminum alloys under complex stress states and optimized hot processing parameters, proposing a new constitutive equation based on the modified Zerilli–Armstrong model and constructing hot processing maps. Ashtiani [8] investigated the influence mechanism of strain on the activation energy and constitutive parameters of 2030 aluminum alloys, establishing a strain-dependent Arrhenius-type constitutive model. The results show that strain significantly affects the activation energy Q and material constants, demonstrating the necessity for strain compensation.

Studies on the thermal deformation rheological behavior of numerous aluminum alloys have shown that the temperature and strain rate are key parameters influencing their thermal deformation behavior. Additionally, micro-mechanisms play an important role: precipitates may hinder dislocation movement during the thermal deformation of aluminum alloys, while the dissolution of precipitates at high temperatures leads to softening.

Currently, there are many studies on constitutive models for describing the flow stress of aluminum alloys, each with its own advantages. The Johnson–Cook model is suitable for wide strain rate ranges, but its independent assumptions of thermal softening and strain rate hardening deviate from actual coupling effects; modified versions improve accuracy by introducing temperature–strain rate interaction terms. The Arrhenius model, based on thermal activation theory, requires coupling with the Zener–Hollomon parameter to describe coupling effects, while the strain-compensated Arrhenius model is more suitable for alloys with significant dynamic softening.

However, the thermal deformation behavior of 5383 aluminum alloys has only been roughly described in a few studies, and a specific constitutive model for this alloy is lacking, especially regarding flow behavior under complex stress states. There is a lack of research on the thermal deformation of 5383 aluminum alloys with different Mg contents, and the existing Johnson–Cook model does not account for the influence of the thermal stability of Mg-enriched phases in AA5383 on flow stress, leading to large simulation errors [9]. The magnesium content plays a crucial role in determining the thermal deformation behavior and microstructural evolution of 5383 aluminum alloys. However, current research lacks comprehensive investigations into the thermal deformation behavior of 5383 alloys with varying Mg contents, leaving a theoretical gap in understanding the influence mechanisms of the Mg content on thermal processing characteristics.

This study systematically investigates the high-temperature deformation behavior of 5383 alloys with different Mg compositions. We establish multi-factor coupled constitutive models to elucidate the temperature–stress–strain correlations and develop a three-dimensional thermal processing diagram to identify optimal thermal forming parameters (deformation temperature and strain rate) under various strain conditions. These findings provide critical theoretical foundations for precision control in the thermal processing of 5xxx aluminum alloys, particularly establishing novel pathways for the synergistic enhancement of microstructural and mechanical properties through Mg content regulation.

## 2. Materials and Methods

**Figure 1:**
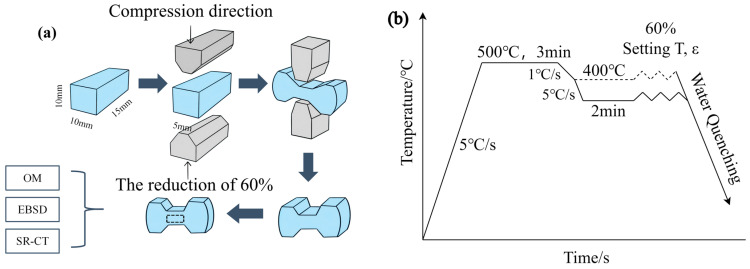
Plane strain compression test method (**a**) and experimental process (**b**).

The experimental 5383 aluminum alloys were provided by ALG Aluminium Inc. (Nanning, China), and the specific compositions of the three alloy samples are listed in Table 1. Isothermal constant strain rate compression tests were performed using a Gleeble-3500 thermo-mechanical simulator, which was provided by Dynamic Systems Inc. (New York, NY, USA), and the plane strain compression methodology and experimental procedure are illustrated in Figure 1. Prior to testing, specimens were coated with lubricant and layered with graphite sheets to minimize friction and ensure uniform deformation. Specimens were heated uniformly to 500 °C at a rate of 5 °C/s, held for 3 min, and then cooled to target temperatures (500 °C, 440 °C, 380 °C, 320 °C, 260 °C, and 200 °C) with a two-minute stabilization period. Plane strain compression was subsequently conducted at each temperature using strain rates of 0.05 s^−1^, 0.5 s^−1^, and 5 s^−1^, achieving a consistent deformation rate of 60%. To realistically simulate hot rolling conditions, differentiated cooling rates were applied: 1 °C/s above 400 °C and 5 °C/s below 400 °C. Flow stress data under various temperature–strain rate conditions were automatically recorded during deformation for subsequent analysis. The deformed specimens were quenched in liquid nitrogen to effectively simulate the water quenching process and preserve the microstructure of thermal deformation.

Compressed specimens were sectioned longitudinally, with the central regions polished and etched for metallographic examination using a Leica DMI 5000 M optical microscope (Leica, Wetzlar, Germany), and microstructural characterization was performed via a Hitachi S-3400N scanning electron microscope (Hitachi, Tokyo, Japan). Synchrotron radiation X-ray computed tomography (SR-CT) was used to investigate the three-dimensional morphology and distribution of Fe/Mn intermetallic compounds. The density contrast between the matrix and iron-rich phases enabled the precise reconstruction of their spatial distribution through differential X-ray attenuation. A combination of analytical techniques (OM, SR-CT, and EBSD) were employed to systematically examine the Mg-content-dependent microstructural characteristics under varying deformation parameters. The SR-CT experiments were conducted at the BL13W1 beamline of the Shanghai Synchrotron Radiation Facility (SSRF). The experimental conditions that SSRF can provide are listed in Table 2. The EBSD experiments were observed using a Hitachi S-3400N scanning electron microscope (Hitachi, Tokyo, Japan), and the deformed microstructure was analyzed with AztecCrystal software (Aztec 5.4 SP1) from Oxford Company (Oxford, UK). Comparative analysis of microstructural evolution patterns was conducted to elucidate the influence of the Mg content on hot working behavior.

## 3. Results and Discussion

### 3.1. Deformation Behavior Analysis

**Figure 2:**
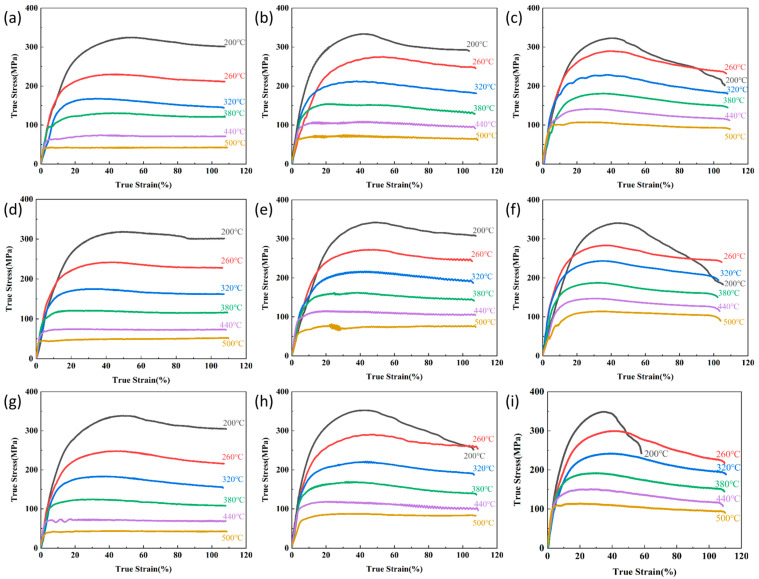
True stress–strain curves of 5383 aluminum alloy with Mg contents of (**a**–**c**) 4.0, (**d**–**f**) 4.5, and (**g**–**i**) 5.0 and strain rates of (**a**,**d**,**g**) 0.05 s^−1^, (**b**,**e**,**h**) 0.5 s^−1^, and (**c**,**f**,**i**) 5 s^−1^. (s^−1^ is the unit for strain rate).

The thermal simulation experiments for the flow stress curves are shown in Figure 2, which illustrate variations in the Mg content, temperature, and strain rate. The curves show a rapid initial stress increase due to dominant work hardening. Under external loading, accelerated dislocation multiplication forms high-density dislocation cell structures, and the obstruction of subsequent dislocation motion leads to sharp rises in deformation resistance [10]. As deformation progresses, accumulated stored energy activates dynamic recovery (DRV) processes through dislocation climb and annihilation. The rearrangement of dislocations into lower-energy configurations reduces entanglement density, partially counteracting work hardening effects and decelerating stress growth. Under high-temperature/low-strain-rate conditions, enhanced thermal activation enables extensive dislocation reorganization through dynamic recrystallization (DRX). During DRX, strain-free grains nucleate, significantly reducing deformation resistance through pronounced softening [11]. This transition from DRV to DRX-dominated mechanisms results in substantially slower stress accumulation rates.

Post-peak stress behavior exhibits three distinct patterns: (1) stress stabilization under continued strain, indicating dynamic equilibrium between work hardening and softening (predominant at high temperatures/low strain rates, e.g., 500 °C/0.05 s^−1^); (2) flow stress reduction until deformation completion, suggesting sustained dominance of dynamic softening over hardening or microcrack-induced stress relaxation; and (3) yield fracture termination.

Notably, the flow curves in Figure 2 still show a gradual transition to steady-state flow at high temperatures, which is characteristic of partial DRX occurring alongside dominant DRV.

**Figure 3:**
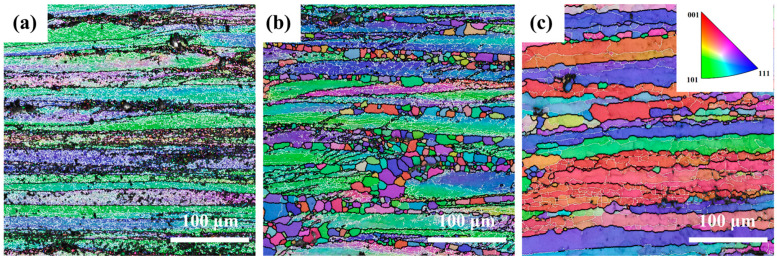
IPF images of Mg4.0-5383 Al alloy at different temperatures and a strain rate of 0.05 s^−1^. (**a**) Temperature of 320 °C; (**b**) 380 °C; and (**c**) 500 °C.

**Figure 4:**
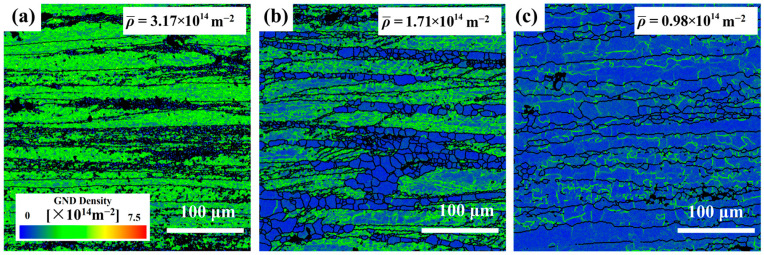
GND images of Mg4.0-5383 Al alloy at different temperatures and a strain rate of 0.05 s^−1^. (**a**) Temperature of 320 °C; (**b**) 380 °C; and (**c**) 500 °C.

Figure 3 shows inverse pole figure (IPF) maps of the Mg4.0-5383 alloy deformed at 0.5 s^−1^ under 320 °C, 380 °C, and 500 °C (from left to right). Black and white lines indicate high-angle (>15°) and low-angle (2–15°) grain boundaries, respectively. Geometrically necessary dislocation (GND) density maps derived from EBSD data (Figure 4) highlight localized strain accumulation zones (bright green regions) with significant orientation gradients and high stored energy.

The recrystallization temperature of the 5383 aluminum alloy is ~260 °C. During deformation at 320 °C, the microstructure primarily consists of elongated deformed grains containing numerous subgrain boundaries (indicated by white lines), with only a few recrystallized grains emerging along grain boundaries. The extensive bright green regions in the GND density maps (Figure 4) reveal exceptionally high residual dislocation density, confirming the dominant work hardening mechanisms and significant stored strain energy at this temperature. At 380 °C, the microstructure exhibits a mixed morphology of recovered deformed grains and larger recrystallized grains. The reduced residual dislocation density suggests the activation of dynamic recovery (DRV) processes, where dislocation climb and rearrangement reduce entanglement density, thereby mitigating work hardening effects. At 500 °C, the microstructure demonstrates characteristic continuous dynamic recrystallization (CDRX) features, with serrated grain boundaries and intragranular recrystallization nuclei. Simultaneously, geometrically dynamic recrystallization (GDRX) is presented as brick-like grain structures. The alignment of bright green GND density patterns with subgrain boundaries indicates enhanced dislocation reorganization into dislocation cells at higher temperatures. These cells progressively evolve into high-angle grain boundaries through dislocation absorption, ultimately forming recrystallized grains and inducing dynamic softening [12].

Table 3 shows that at constant strain rates, the peak flow stress of 5383 alloys decreases significantly with increasing temperature across all Mg contents. This temperature dependence is attributed to three primary mechanisms: (1) Enhanced atomic thermal motion at elevated temperatures weakens interatomic bonding and activates additional slip systems, thereby reducing deformation energy requirements. (2) Thermal activation promotes dislocation rearrangement through dynamic recovery, while accelerated grain boundary migration facilitates dynamic recrystallization nucleation and growth, collectively lowering dislocation density. (3) The dissolution of Mg-rich Al_8_Mg_5_ precipitates above critical temperatures reduces dislocation pinning effects, further decreasing flow stress [13].

**Figure 5:**
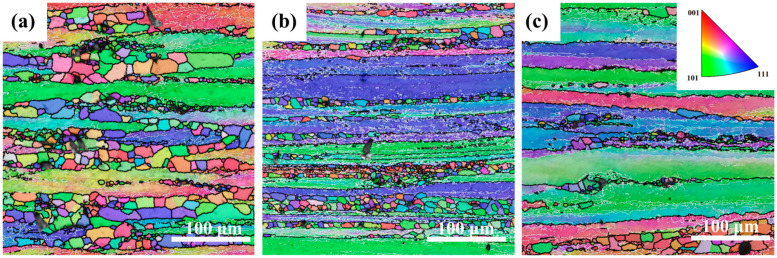
IPF images of Mg4.0-5383 aluminum alloy at 440 °C with different strain rates: (**a**) 0.05 s^−1^; (**b**) 0.5 s^−1^; and (**c**) 5 s^−1^.

**Figure 6:**
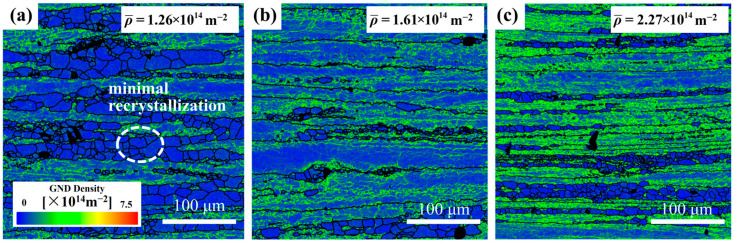
GND images of Mg4.0-5383 aluminum alloy at 440 °C with different strain rates: (**a**) 0.05 s^−1^; (**b**) 0.5 s^−1^; and (**c**) 5 s^−1^.

Figure 5 displays the microstructural evolution of the Mg5.0 alloy at 440 °C under increasing strain rates (left to right). All conditions exhibit elongated banded structures with abundant subgrain boundaries (white lines), confirming DRV dominance. At 0.05 s^−1^, numerous recrystallized grains are observed. With the strain rate increasing to 0.5 s^−1^, the band thickness decreases while retaining smaller recrystallized grains. At 5 s^−1^, further band refinement occurs with extensive subgrain boundaries and minimal recrystallization. Corresponding GND density maps (Figure 6) demonstrate the progressive expansion of high-dislocation-density regions (bright green) with an increasing strain rate, indicating accumulated residual dislocations. This phenomenon is attributed to the rapid accumulation of high stress within materials due to high strain rates. When the stress exceeds the critical value for dislocation multiplication, even if the nucleation process is limited by time constraints, mechanisms such as double-crossing slip and climb-up can still operate efficiently under high stress, rapidly increasing the dislocation density through multiplication rather than nucleation. Additionally, at high strain rates, dislocations move at inconsistent speeds, making it easier for dislocation accumulation to occur and for complex entanglement structures to form at obstacles like grain boundaries and second-phase particles. These structures hinder further dislocation movement and annihilation, leading to dislocations being retained in the material.

At constant deformation temperatures, peak flow stress increases substantially with the strain rate with various Mg contents. This strain rate sensitivity originates from two synergistic effects: (1) Reduced deformation duration limits dislocation mobility and the completion of DRV, preserving work-hardened structures. (2) Insufficient time for DRX nucleation and growth prior to deformation termination suppresses dynamic softening mechanisms. These combined effects result in sustained high dislocation density and consequently elevated flow stress [14,15].

Across all temperature–strain rate conditions, peak stress increases with the Mg content. This peak stress corresponds to the balance between work hardening and dynamic softening. The solid solution strengthening effect arises from the atomic size mismatch between Mg solutes and Al matrix atoms, which induces significant lattice distortion. A higher Mg content increases this distortion, creating stronger dislocation barriers and consequently higher flow stress. Additionally, Mg segregation leads to dislocation pinning via Cottrell atmospheres, effectively suppressing the dislocation climb and annihilation processes necessary for DRV. These combined effects prolong the dominance of work hardening while inhibiting DRX, thereby driving the increase in peak stress with the Mg content.

Notably, post-peak stress reduction becomes more pronounced with higher Mg concentrations. This phenomenon correlates with Mg’s dual role in solid solution strengthening and its interaction with iron-rich phases (Al_6_(Fe,Mn)). An elevated Mg content increases residual dislocation density while modifying the size and distribution of iron-rich phases. The resulting dislocation network provides abundant nucleation sites for DRX through orientation gradient accumulation at grain boundaries. Concurrently, enhanced dislocation pinning at secondary phases creates localized stress concentrations that accelerate DRX initiation. These coordinated effects promote intensive dynamic softening that overcomes prior work hardening, leading to significant stress reduction during the post-peak stress stage.

### 3.2. Establishment of Constitutive Equations

The Arrhenius model [16], which is widely used to describe the relationship between the strain rate, deformation temperature, and flow stress under high-temperature deformation and to characterize the stress–strain curve as increasing and then decreasing, can be expressed for all stress conditions as follows:

(1) $$\dot{\varepsilon }=A[\sinh\left(\alpha \sigma \right){]}^{n}exp(-\frac{Q}{RT})$$

It can be further categorized for different strains as follows:

(2) $$\dot{\varepsilon }=A_{1}\sigma^{n_{1}}exp(-\frac{Q}{RT})\;(\alpha \cdot \sigma \;<\;0.8)$$

(3) $$\dot{\varepsilon }=A_{2}exp(\beta \sigma )exp(-\frac{Q}{RT})\;(\alpha \cdot \sigma \;>\;1.2)$$

The Zener–Hollomon parameter (referred to as the Z-parameter) proposed by Zener and Hollomon [17] can characterize the effect of the deformation temperature and strain rate on strain stress.

(4) $$Z=\dot{\varepsilon }exp\left(\frac{Q}{RT}\right)$$

where $A,\;A_{1},\;A_{2},\;n,\;n_{1},\;\alpha ,\;and\;\beta$ are the material parameters, and α = β/$n_{1};\;\dot{\varepsilon }$ is the strain rate (s^−1^); σ is the flow stress (MPa); R is the gas constant (kJ/(mol·K)); Q is the activation energy of deformation (kJ/mol); and T is the activation energy of deformation (K). By using the three Arrhenius model equations, it is possible to comprehensively and accurately predict flow stresses of thermal deformation.

Substituting Equation (4) into Equation (1) yields

(5) $$Z=A[sinh(\alpha \sigma ){]}^{n}$$

Taking logarithms on both sides of Equations (1)–(3) and (5), the following four equations are obtained:

(6) $$ln\dot{\varepsilon }=lnA+nln\left[sinh\left(\alpha \sigma \right)\right]-\frac{Q}{RT}$$

(7) $$ln\dot{\varepsilon }=lnA_{1}+n_{1}ln\sigma -\frac{Q}{RT}$$

(8) $$ln\dot{\varepsilon }=lnA_{2}+\beta \sigma -\frac{Q}{RT}$$

(9) $$lnZ=lnA+n_{3}ln[sinh(\alpha \cdot \sigma )]$$

Equation (6) is partially differentiated with respect to 1/T, and the thermal deformation activation energy equation is obtained using the final calculation:

(10) $$Q=R{\left[\frac{\partial ln\dot{\varepsilon }}{\partial ln\left[sinh\left(\alpha \sigma \right)\right]}\right]}_{T}{\left[\frac{\partial ln\left[sinh\left(\alpha \sigma \right)\right]}{\partial \left(\frac{1}{T}\right)}\right]}_{\dot{\varepsilon }}$$

**Figure 7:**
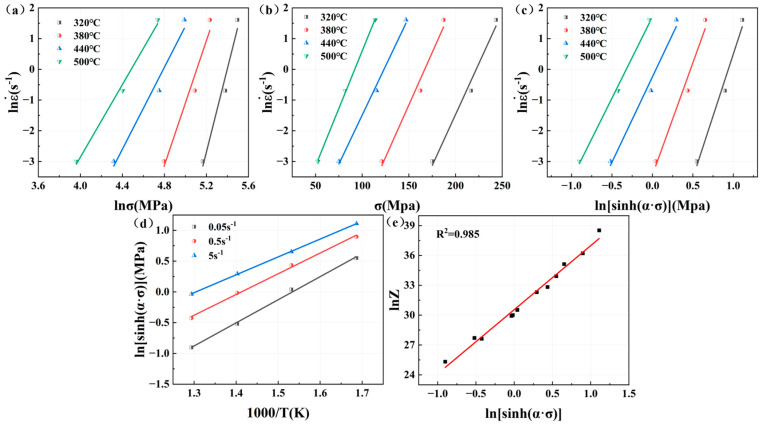
Relationship curves among peak flow stress, strain rate, and deformation temperature of Mg4.0-5383 aluminum alloys: (**a**) $ln\dot{\varepsilon }-ln\sigma$; (**b**) $ln\dot{\varepsilon }-\sigma$; (**c**) $ln\dot{\varepsilon }-ln\left[sinh\left(\alpha \sigma \right)\right]$; (**d**) $ln\left[sinh\left(\alpha \sigma \right)\right]-1/T$ and (**e**) $lnZ-ln\left[sinh\left(\alpha \sigma \right)\right]$.

$n_{1}$ represents the ln$\dot{\varepsilon }$ − lnσ curve slope of the average value, and β represents the ln$\dot{\varepsilon }$−σ curve slope of the average value, respectively. For ln$\dot{\varepsilon }$ − lnσ and ln$\dot{\varepsilon }$ − σ relationship curves, as shown in Figure 7a,b, an $n_{1}$ value of 9.0871 and a β value of 0.06834 can be obtained, with α = β/$n_{1}$ = 0.007521.

From Equation (9), it can be seen that there is a linear relationship between $ln\dot{\varepsilon }$ and $ln\left[sinh\left(\alpha \sigma \right)\right]$; $ln\left[sinh\left(\alpha \sigma \right)\right]$ − 1/T also has a linear relationship. n is the average value of the slope of the $ln\dot{\varepsilon }$ − $ln\left[sinh\left(\alpha \sigma \right)\right]$ curve; K is the average value of the slope of $ln\left[sinh\left(\alpha \sigma \right)\right]$ − 1/T curve. The relationship curve of $ln\dot{\varepsilon }$ − $ln\left[sinh\left(\alpha \sigma \right)\right]$ is shown in Figure 7c, and the value of n is 6.5501; the curve of $ln\left[sinh\left(\alpha \sigma \right)\right]$ − 1/T is shown in Figure 7d, and the value of K is 3.3424. Combined with the value of n, Q = RnK = 182.01 kJ/mol. By substituting the value of Q into Equation (4) to calculate the value of Z with different deformation parameters, such as $lnZ-ln\left[sinh\left(\alpha \sigma \right)\right]$, as shown in Figure 7e, we can get the value of A, which is 1.8567 × 1013. In summary, the constitutive equation of Mg4.0-5383 aluminum alloy is

$$\dot{\varepsilon }=1.86\times 10^{13}{\left[sinh(0.0075\sigma )\right]}^{6.55}exp(-182013/RT)$$

**Figure 8:**
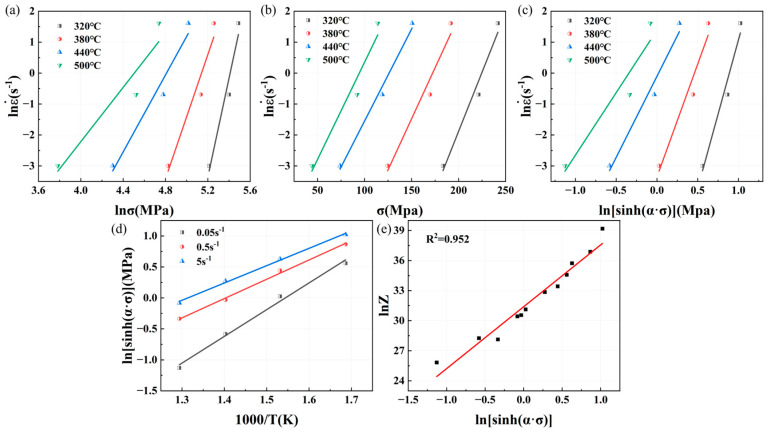
Relationship curves among peak flow stress, strain rate, and deformation temperature of Mg4.5-5383 aluminum alloys: (**a**) $ln\dot{\varepsilon }-ln\sigma$; (**b**) $ln\dot{\varepsilon }-\sigma$; (**c**) $ln\dot{\varepsilon }-ln\left[sinh\left(\alpha \sigma \right)\right]$; (**d**) $ln\left[sinh\left(\alpha \sigma \right)\right]-1/T$ and (**e**) $lnZ-ln\left[sinh\left(\alpha \sigma \right)\right]$.

**Figure 9:**
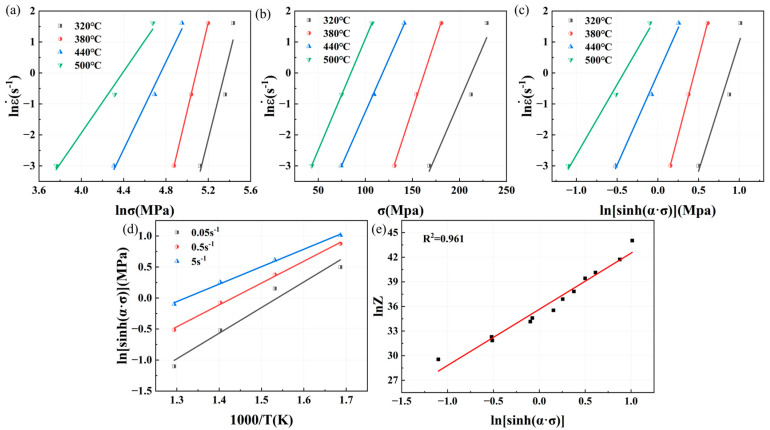
Relationship curves among peak flow stress, strain rate, and deformation temperature of Mg5.0-5383 aluminum alloys: (**a**) $ln\dot{\varepsilon }-ln\sigma$; (**b**) $ln\dot{\varepsilon }-\sigma$; (**c**) $ln\dot{\varepsilon }-ln\left[sinh\left(\alpha \sigma \right)\right]$; (**d**) $ln\left[sinh\left(\alpha \sigma \right)\right]-1/T$ and (**e**) $lnZ-ln\left[sinh\left(\alpha \sigma \right)\right]$.

Similarly, the relationship curves between peak stress, strain rate, and deformation temperature of Mg4.5-5383 aluminum alloys and Mg5.0-5383 aluminum alloys can be obtained as shown in Figure 8 and Figure 9. And then we can determine that the $n_{1}$ value of Mg4.5 is 9.1545, β is 0.06618, α is 0.007229, n is 6.5394, K is 3.4081, Q = RnK = 185.2841 kJ/mol, and A is 4.3206 × 1013; additionally, the $n_{1}$ value of Mg5.0 is 9.9986, β is 0.07587, α is 0.007588, n is 7.2109, K is 3.4892, Q = RnK = 209.1821 kJ/mol, and A is 3.0625 × 1015.

The constitutive equation of Mg4.5-5383 aluminum alloys is

$$\dot{\varepsilon }=4.32\times 10^{13}{\left[sinh(0.0072\sigma )\right]}^{6.54}exp(-185284/RT)$$

The constitutive equation of Mg5.0-5383 aluminum alloys is

$$\dot{\varepsilon }=3.06\times 10^{15}{\left[sinh(0.0076\sigma )\right]}^{7.21}exp(-209182/RT)$$

During the thermal processing of 5383 aluminum alloys, deformation resistance increases with a higher Mg content, mainly due to the solid solution hardening effect. This study identifies a threshold at 5 wt.% Mg, where the hot deformation activation energy jumps to 209 kJ/mol. The enhanced solid solution strengthening induces two factors: (1) elevated deformation stress, which promotes serrated grain boundary formation through constrained boundary migration, and (2) stress concentrations along these serrated boundaries, which facilitate void/crack nucleation [18], creating drag effects on dislocation motion. These dual mechanisms suppress dynamic recovery (dislocation rearrangement and annihilation) while promoting DRX initiation. The energy-intensive nature of DRX, which requires new grain boundary formation, directly accounts for the observed increase in activation energy.

### 3.3. Analysis of 3D Thermal Processing Diagrams

A thermal processing map was constructed by superimposing power dissipation and plastic instability maps based on the dynamic materials model (DMM) proposed by Prasad [19].This analytical tool effectively reveals the correlation between microstructural evolution and processing parameters during hot deformation, providing guidance for optimizing thermal processing conditions [20,21]. To characterize the hot workability of 5383 aluminum alloy under varying strains, we extend conventional 2D processing maps to three-dimensional representations incorporating strain evolution. These 3D maps quantitatively visualize material power dissipation and instability domains within the parameter space of temperature, strain rate, and strain.

**Figure 10:**
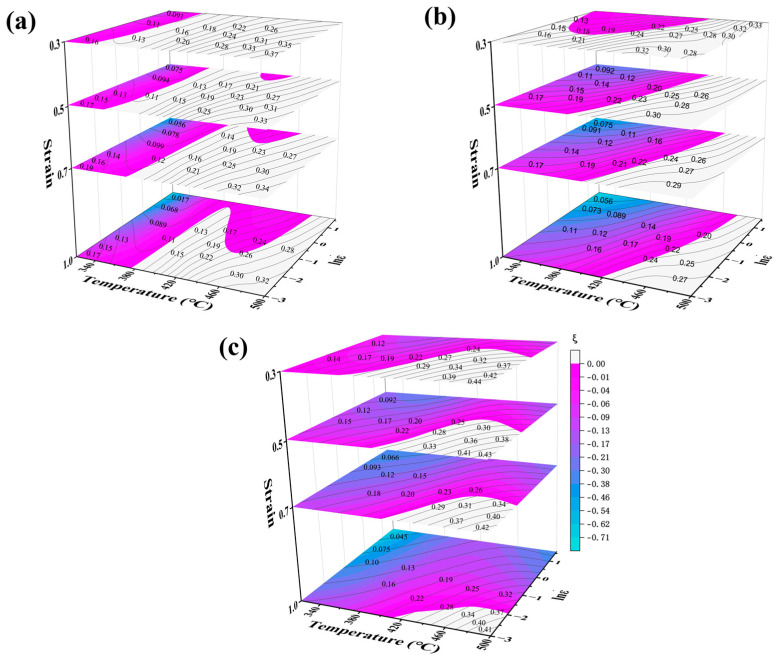
Three-dimensional thermal processing maps of 5383 aluminum alloy with different Mg contents: (**a**) Mg4.0; (**b**) Mg4.5; and (**c**) Mg5.0.

Figure 10 presents the 3D thermal processing maps for the Mg4.0, Mg4.5, and Mg5.0 variants. The black contour lines denote power dissipation efficiency (η%), while the blue-purple gradients indicate instability regions (ξ < 0). The maps encompass complete processing windows: strain (0.3~1.0), temperature (320~500 °C), and strain rate (0.05~5 s^−1^). Figure 10 shows that the safe region for the full-strain hot working of Mg4.0-5383 aluminum alloy is 390~500 °C with a strain rate of 0.05~0.4 s^−1^; for Mg4.5, it is 420~500 °C with a strain rate of 0.05~0.4 s^−1^; and for Mg5.0, it is 420~500 °C with a strain rate of 0.05~0.16 s^−1^. The progressive narrowing of stable regions with an increasing Mg content demonstrates enhanced processing sensitivity to strain rate variations.

**Figure 11:**
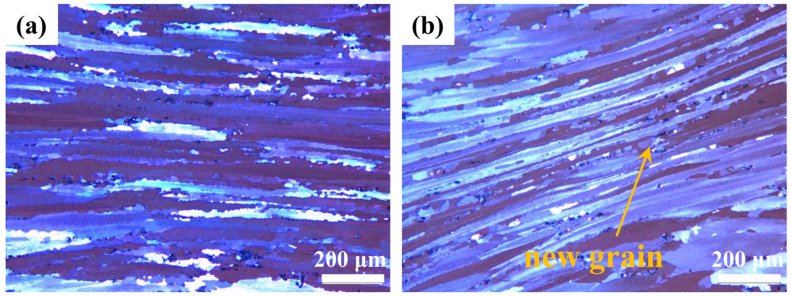
Deformation structure in safe zones of Mg4.0-5383 aluminum alloys: (**a**) 440 °C/0.05 s^−1^; (**b**) 500 °C/0.05 s^−1^.

**Figure 12:**
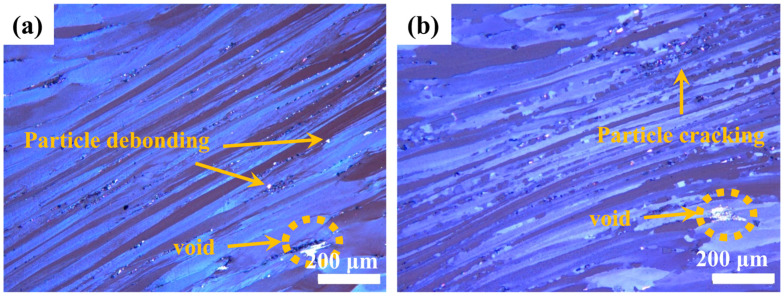
Deformation structure in unstable zones of Mg4.0-5383 aluminum alloys: (**a**) 320 °C/5 s^−1^; (**b**) 440 °C/5 s^−1^.

As shown in Figure 11, the safe processing zones exhibit characteristic dynamically recovered structures featuring elongated fibrous grains with serrated grain boundaries. DRX increases the continuous chain-like structure of fine equiaxed grains nucleated through the grain boundary. In contrast, Figure 12 reveals microstructural instabilities characterized by localized shear bands and void formation through iron-rich phase debonding. These defect-containing regions reduce thermal performance and are thus unsuitable for thermal processing.

The three-dimensional thermal processing maps of 5383 aluminum alloys with varying Mg contents reveal several results. Firstly, at a constant strain rate, increasing the temperature or decreasing the strain rate markedly enhances the power dissipation factor. In high-temperature environments, the resistance to dislocation motion is diminished, allowing dislocations to continuously consume energy through slip rearrangement or annihilation. Moreover, elevated temperatures facilitate DRX and eliminate microscopic defects, such as dislocation plugging, thereby further improving energy dissipation efficiency. Low strain rates provide ample time for DRX and grain growth. Secondly, the peak power dissipation gradually decreases with increasing strain. This is attributed to the increased dislocation pile-up, which hampers energy consumption through rearrangement or annihilation and consequently reduces the energy dissipation efficiency of the DRV and DRX processes. Thirdly, the expansion of the destabilization region with increasing strain is closely linked to DRX. Under high-strain conditions, the stored energy generated by dislocation accumulation can serve as a driving force for DRX. However, abnormal grain growth and localized softening of recrystallized grains can significantly compromise the thermal processing stability of the material. Additionally, increased dislocation buildup can lead to localized stress concentrations, which contribute to the formation of microcracks and voids, thereby accelerating the material destabilization process.

According to the three-dimensional thermal processing maps of 5383 aluminum alloys with different Mg contents, the following statements can be made: First, the safe processing region diminishes as the Mg content increases. The safe processing region of Mg4.0-5383 encompasses the largest area, accounting for more than half of the total area up to a strain of 0.7. In contrast, the safe processing region of Mg5.0-5383 occupies only 8% of the area at a strain of 1.0. Second, the peak power dissipation for Mg4.0 and Mg5.0-5383 aluminum alloys is consistently located in the high-temperature, low-strain-rate region (near 500 °C and 0.05 s^−1^) across different strains. However, for Mg4.5-5383 aluminum alloys, the peak power dissipation appears in this region only when the strain reaches a value of 0.5 or higher. Third, under the same deformation conditions, the peak power dissipation increases with a rising Mg content, with values of 35% for Mg4.0-5383, 39% for Mg4.5-5383, and 47% for Mg5.0-5383.

**Figure 13:**
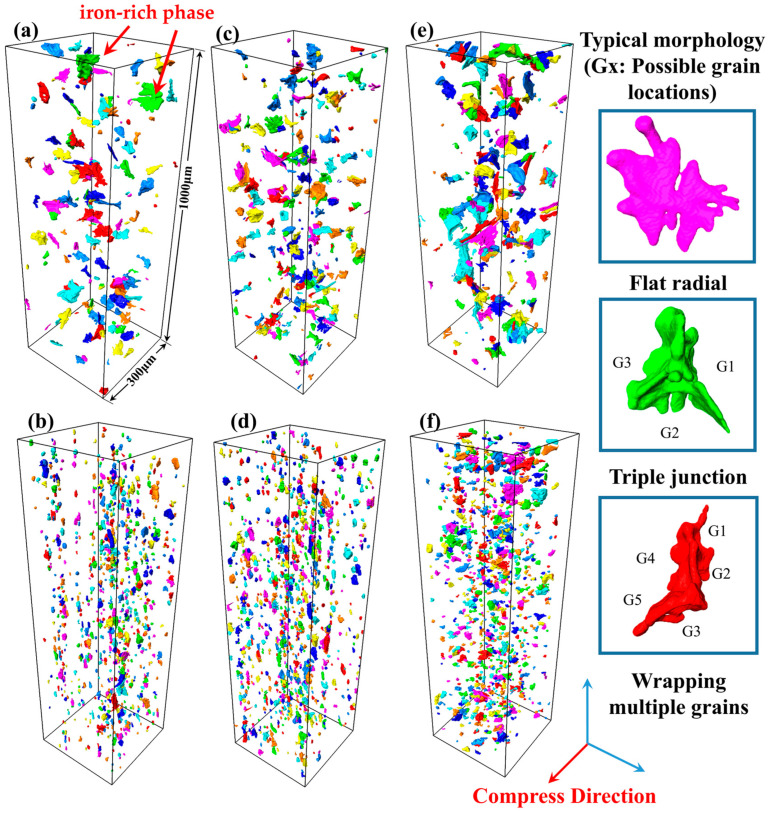
SR-CT of 5383 aluminum alloys: homogeneous state on upper side and H116 rolled state on lower side. (**a**,**b**) Mg4.0; (**c**,**d**) Mg4.5; and (**e**,**f**) Mg5.0.

In order to visually and quantitatively analyze the effect of Mg on the thermal processing of 5383 aluminum alloys, the 3D morphology and distribution of Fe/Mn intermetallic compounds are investigated using SR-CT. Figure 13a,c,e show the 3D morphology and distribution of the iron-rich phase with the increase in the Mg content before thermal deformation. It can be observed that most of the iron-rich phases before deformation are distributed as irregular particles, and the volumes of the iron-rich phases are slightly larger in the three typical areas: flat and dendritic outward radiation, a triangular distribution at the junction of the three grains, and a complex structure wrapped around multiple grains. The average size and volume of the Al_6_(Fe,Mn) iron-rich phases increase with the Mg content.

Figure 13b,d,f show the changes in the 3D morphology and distribution of the iron-rich phase with the increase in the Mg content after compression deformation. After roll deformation, the iron-rich phase is distributed along the rolling direction according to a certain orientation, and the crushed iron-rich phase is distributed along the grain boundaries between the narrow and long bands of deformed grains. At this time, there is no longer a larger iron-rich phase in Mg4.0, and the morphology of the iron-rich phase is close to smaller spherical particles scattered near the long grain boundaries. But with an increase in the Mg content, the larger iron-rich phase can still be observed in Mg5.0. The average size and unit volume of the iron-rich phase increase with the increase in the Mg content after deformation, the same as what was observed before deformation.

It can be seen that the Mg content has a significant effect on the morphology of the iron-rich phase and the hot working properties of the alloy. In the casting process, for Mg-containing iron-rich intermetallic compounds, magnesium is an essential component in both the crystallization nucleation stage and the growth stage. During the nucleation stage, as an alloying element, magnesium may participate in the formation of iron-rich phase nuclei, promoting nucleation by reducing the nucleation energy barrier. In the growth stage, magnesium continues to be incorporated into the lattice of iron-rich phases as a constituent element or regulates crystal growth kinetics by influencing interfacial diffusion rates. In this process, magnesium is not only a chemical component of Mg-containing iron-rich phases, but its concentration may also affect the precipitation path, size, and distribution of these phases, thereby significantly impacting the properties of aluminum alloys [22], and the high Mg content leads to the saturation of Mg elements in the matrix α-Al, which significantly increases the diffusion resistance of Mg elements from the iron-rich phase to the matrix. This diffusion inhibition effect hinders the dissolution of the Fe-rich phase in the homogenization process, which ultimately leads to the increase in its size with the increase in the Mg content. During thermal deformation, these large, hard iron-rich phases increase the stress concentration and severely limit the metal flow properties. Therefore, compared to the other two aluminum alloys with lower Mg contents, higher temperatures (≥420 °C) and lower strain rates (≤0.16s^−1^) are required to ensure the deformation of the Mg5.0-5383 aluminum alloy, which directly results in the smallest processing safety region over the full strain range in its 3D thermal processing diagram. So, the safe deformation region decreases with the increase in the Mg content.

**Figure 14:**
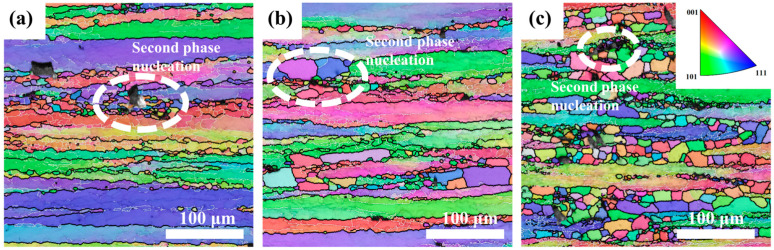
IPF images of different Mg contents at a temperature of 440 °C and a strain rate of 0.05 s^−1^: (**a**) Mg4.0; (**b**) Mg4.5; and (**c**) Mg5.0.

**Figure 15:**
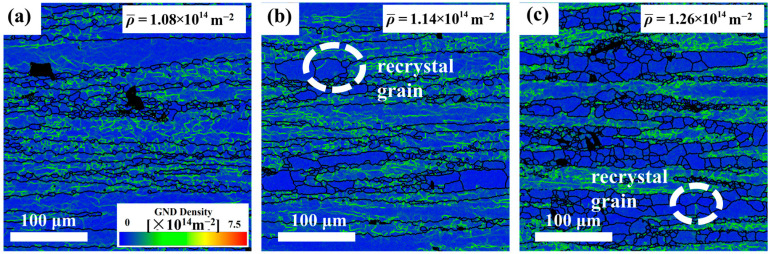
GND images of different Mg contents at a temperature of 440 °C and a strain rate of 0.5 s^−1^: (**a**) Mg4.0; (**b**) Mg4.5; and (**c**) Mg5.0.

Figure 14 shows the IPF maps of 5383 alloys with varying Mg contents deformed at 440 °C/0.05 s^−1^. All variants exhibit elongated banded structures, with Mg5.0 showing significantly higher recrystallized grain density. The white boxes in Figure 14a–c highlight recrystallization nucleation around secondary phases, which is observed across all compositions but is the most pronounced in Mg5.0. As shown in Figure 15, increasing the Mg content elevates the residual dislocation density while simultaneously enlarging the Fe-rich phase size and volume fraction. This dual effect creates abundant nucleation sites for recrystallization, explaining the enhanced DRX observed in Mg5.0 (Figure 15c) under these deformation conditions. The intensified microstructural evolution (dislocation rearrangement/grain boundary migration) consumes additional energy, so Mg5.0 exhibits the highest value of power dissipation in the thermal processing diagrams.

## 4. Conclusions

(1) Mg addition (4.0–5.0 wt.%) elevates the peak flow stress of 5383 aluminum alloys by intensifying lattice distortion, dislocation blocking, and second-phase interactions. Post-peak stress decreases with a higher Mg content, attributed to enhanced dynamic softening mechanisms.
(2) The constitutive equation for the peak flow stress of 5383 aluminum alloys, which was constructed based on the Arrhenius model, reveals that Mg-induced solid solution strengthening increases the thermal activation energy from 182 kJ/mol at 4 wt.% to 209 kJ/mol at 5 wt.% Mg, reflecting suppressed DRV and increased DRX via solute dislocation interactions. The promoted DRX at a higher Mg content occurs due to nucleation through an elevated residual dislocation density, enabling energy-efficient grain boundary restructuring.
(3) Increased Mg expands instability domains, narrowing safe processing windows from Mg4.0 (390~500 °C/0.05~0.4 s^−1^) to Mg5.0 (420~500 °C/0.05~0.16 s^−1^). The main reason for this is that iron-rich phase coarsening exacerbates localized dislocation accumulation; therefore, higher temperatures and lower strain rates are needed for stability. The results show that the relative high Mg content can obviously affect dislocation dynamics, DRX kinetics, and processing stability, thus providing a microstructure–property map for high-Mg 5xxx aluminum alloys.

## Figures and Tables

**Table 1:** Chemical composition of experimental alloys (mass fraction, %).

Composition	Mass Fraction, %					
	Mg	Mn	Si	Cr	Fe	Al
Mg4.0-5383	4.11	0.68	0.06	0.09	0.14	Bal.
Mg4.5-5383	4.49	0.68	0.07	0.08	0.16	Bal.
Mg5.0-5383	5.03	0.70	0.09	0.07	0.19	Bal.

**Table 2:** The experiment parameters used in the SSRF.

Experimental Parameter	Specific Value
Radiation Mode	Polychromatic Radiation
X-ray Energy (keV)	19
Resolution (μm)	0.65
Exposure Time (s)	0.5
Total Number of Projection Images	1200
Distance Between Sample and Detector (cm)	19

**Table 3:** Peak flow stress values for 5383 aluminum alloys with varying Mg contents (MPa).

Temperature /°C	Strain Rate of 0.05 s^−1^			Strain Rate of 0.5 s^−1^			Strain Rate of 5 s^−1^		
	Mg4.0	Mg4.5	Mg5.0	Mg4.0	Mg4.5	Mg5.0	Mg4.0	Mg4.5	Mg5.0
200	324.54	318.39	338.16	333.45	341.97	352.17	322.75	340.57	348.57
260	230.36	241.81	247.94	274.94	272.27	290.21	289.82	283.41	299.52
320	167.83	175.11	183.37	212.17	216.59	221.31	228.94	243.34	241.77
380	130.99	120.98	124.43	154.23	162.21	169.44	180.72	187.14	191.67
440	74.28	74.66	73.78	109.16	115.48	118.8	141.29	146.84	150.54
500	43.02	52.52	43.934	74.77	81.634	87.60	107.17	113.94	114.06

## Data Availability

The original contributions presented in this study are included in the article. Further inquiries can be directed to the corresponding authors.
